# Myosin filament-based regulation of the dynamics of contraction in heart muscle

**DOI:** 10.1073/pnas.1920632117

**Published:** 2020-03-27

**Authors:** Elisabetta Brunello, Luca Fusi, Andrea Ghisleni, So-Jin Park-Holohan, Jesus G. Ovejero, Theyencheri Narayanan, Malcolm Irving

**Affiliations:** ^a^Randall Centre for Cell and Molecular Biophysics, School of Basic and Medical Biosciences, King’s College London, SE1 1UL London, United Kingdom;; ^b^British Heart Foundation Centre of Research Excellence, King’s College London, SE1 1UL London, United Kingdom;; ^c^European Synchrotron Radiation Facility, 38043 Grenoble, France

**Keywords:** heart muscle, myosin motor, muscle regulation, myosin-binding protein C

## Abstract

Myosin-based mechanisms are increasingly recognized as supplementing their better-known actin-based counterparts to control the strength and time course of contraction in both skeletal and heart muscle. Here we use synchrotron small-angle X-ray diffraction to determine the structural dynamics of local domains of the myosin filament during contraction of heart muscle. We show that, although myosin motors throughout the filament contribute to force development, only about 10% of the motors in each filament bear the peak force, and these are confined to the filament domain containing myosin binding protein-C, the “C-zone.” Myosin motors in domains further from the filament midpoint are likely to be activated and inactivated first in each contraction. Inactivated myosin motors are folded against the filament core, and a subset of folded motors lie on the helical tracks described previously. These helically ordered motors are also likely to be confined to the C-zone, and the associated motor conformation reforms only slowly during relaxation. Myosin filament stress-sensing determines the strength and time course of contraction in conjunction with actin-based regulation. These results establish the fundamental roles of myosin filament domains and the associated motor conformations in controlling the strength and dynamics of contraction in heart muscle, enabling those structures to be targeted to develop new therapies for heart disease.

## Significance

Cardiovascular disease continues to be the leading cause of death worldwide, and is frequently associated with heart failure. Efforts to develop better therapeutics for heart failure have been held back by limited understanding of the normal control of contraction on the timescale of the heartbeat. We used synchrotron X-ray diffraction to determine the dynamic structural changes in the myosin motors that drive contraction in the heart muscle, and show that myosin filament-based control mechanisms determine the time course and strength of contraction, allowing those mechanisms to be targeted for developing new therapies for heart disease.

The pumping action of the heart is driven by rhythmic contractions of its muscular walls. The healthy heart continuously optimizes the strength and time course of contraction by modulating the calcium transient that triggers the heartbeat and the phosphorylation levels of multiple proteins, including components of the myosin and actin filaments that drive contraction, and by direct mechanical feedback (1–4). These signaling pathways alter contractility by changing the structures of the contractile filaments through downstream effector mechanisms that remain poorly understood. For many years attention was focused on actin filament-based regulation and its link to intracellular calcium signaling (1); more recently it became clear, partly by extrapolation from studies on skeletal muscle (5–9), that myosin filament-based regulation also plays an important role. Moreover, myosin-based regulation is perturbed in heart disease (10, 11), and has been increasingly targeted for the development of novel therapies to treat the failing heart (12). Such efforts have however been impeded by limited knowledge about the action of myosin-based regulation on the timescale of the heartbeat: that is, about mechanisms that operate much faster than kinase signaling (3, 4). Two leading candidate mechanisms of this type emerged from studies of skeletal muscle: direct mechanosensing by the myosin filaments (6, 7), and interfilament signaling by myosin binding protein-C (6, 13). Although several studies have suggested that these mechanisms are also present in the heart (2, 14–17), until now it has not been possible to investigate their structural basis in intact heart muscle on the timescale of the heartbeat. Here we exploit recent advances in synchrotron beamlines for high-resolution small-angle X-ray diffraction to determine the structure, function, and dynamics of local domains of the myosin filament in contracting heart muscle with 20-ms time resolution. The results highlight the roles of myosin-based regulation and distinct myosin filament domains in determining the time course of contraction.

## Results and Discussion

### Structural Dynamics of Contraction in Heart Muscle.

**Fig. 1.**
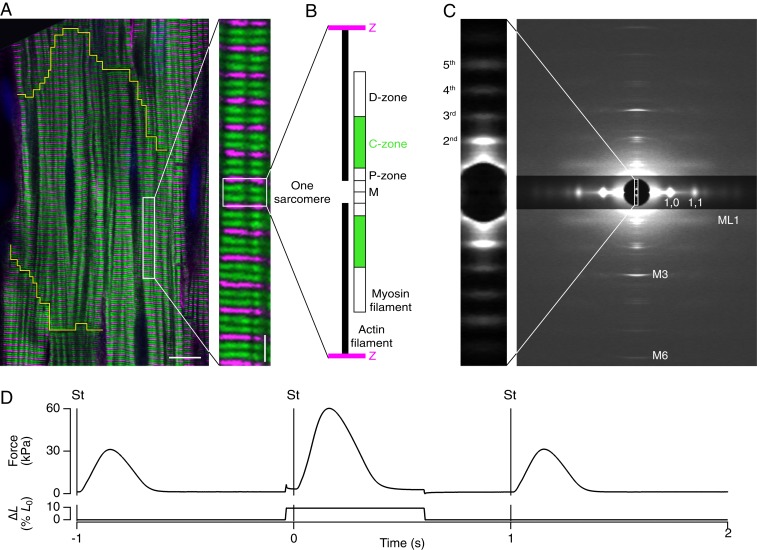
Structural organization and X-ray diffraction from myosin filaments in beating cardiac trabeculae. (*A*) Confocal micrograph of a rat cardiac trabecula stained with anti–α-actinin (magenta) and anti–MyBP-C (green); cardiomyocyte boundary outlined in yellow. (Scale bar 10 μm; *Inset*, 2 μm.) (*B*) Organization of actin (black) and myosin filaments in one sarcomere repeat, indicating the MyBP-C–containing C-zone (green) and the proximal P- and distal D-zones (white); myosin filament midpoint, M; Z-band, Z (magenta). (*C*) Small-angle X-ray diffraction pattern from demembranated trabeculae in relaxing solution at SL 2.15 µm, 27 °C, showing meridional myosin-based reflections M1 to M6, the first myosin layer line (ML1), and the 1,0 and 1,1 equatorial reflections (digitally attenuated). Data added from four trabeculae; total exposure time, 160 ms; detector distance, 1.6 m. (*Inset*) Ultrasmall angle X-ray pattern showing the second-fifth order reflections from the sarcomere repeat; total exposure time, 60 ms; detector distance, 31 m. (*D*) Time course of force and length change as percentage of initial length (% *L*_0_). Trabeculae were stimulated (vertical line, St) continuously at 1 Hz at SL 1.95 µm, 27 °C. Once per minute trabeculae were stretched by 10% *L*_0_ in 5 ms, starting 40 ms before the stimulus. The original length was restored 600 ms after the stimulus.

**Fig. 2.**
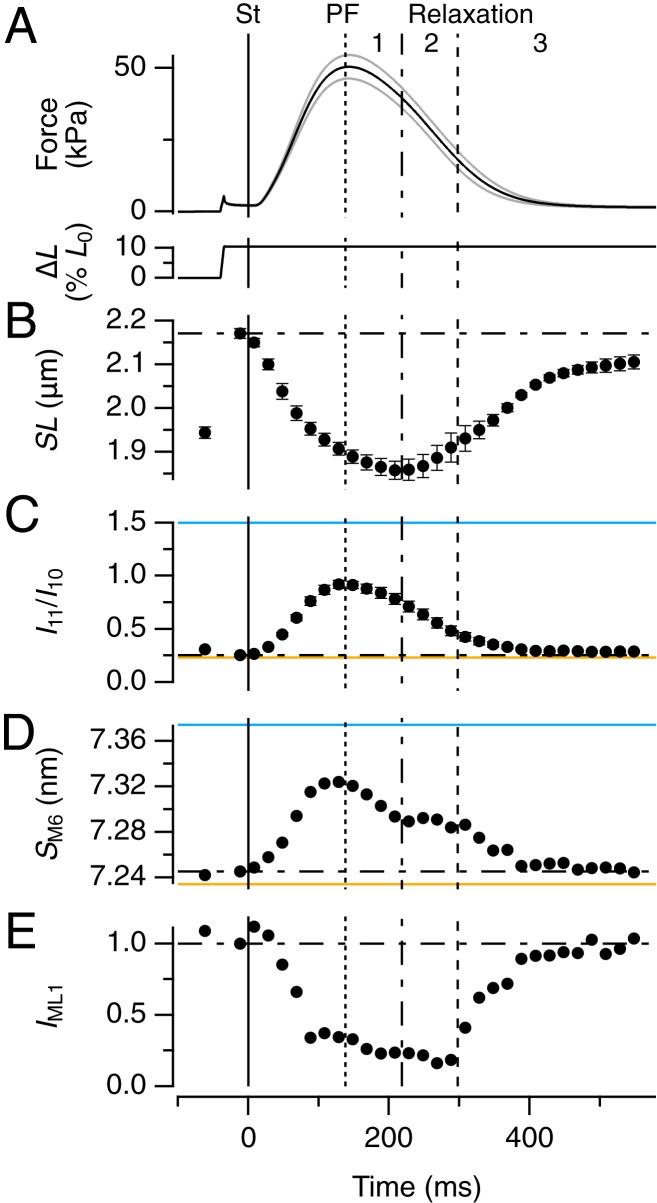
Structural dynamics of myosin motors and filaments during the heartbeat. (*A*) Force and trabecular length change (Δ*L*, expressed as a percentage of initial length [*L*_0_]); vertical continuous line at *t* = 0 indicates the electrical stimulus (St). Vertical dotted, dot-dashed, and dashed lines indicate PF and the end of phase 1 and 2 of relaxation, respectively. Gray traces show ± SEM for force; *n* = 6 trabeculae. (*B*–*E*) Changes in SL (*B*), ratio of equatorial intensities (*I*_11_/*I*_10_, *C*), spacing of M6 reflection (*S*_M6_, *D*) and intensity of ML1 reflection (*I*_ML1_, *E*). Error bars in *B* and *C* are SEM for *n* = 6 trabeculae; data in *D* and *E* added from the same 6 trabeculae. Spatial calibration described in *Materials and Methods*. Horizontal dot-dashed lines indicate the value of each parameter before the stimulus. Horizontal continuous lines in *C* and *D* from two demembranated trabeculae in relaxation at [Ca^2+^] = 1 nM (orange) and during active isometric contraction at [Ca^2+^] = 20 µM (blue), force 95 kPa.

Single trabeculae, consisting of highly uniform three-dimensional (3D) arrays of hundreds of electrically and mechanically coupled heart muscle cells with aligned contractile filaments (Fig. 1*A*) were dissected from the right ventricle of rat heart. The length of each overlapping array of myosin and actin filaments—the sarcomere length (SL) (Fig. 1*B*)—was measured continuously by ultrasmall angle X-ray diffraction (4) (Fig. 1 *C*, *Inset*, and Movie S1). Trabeculae were electrically stimulated once per second (Fig. 1*D*). Once per minute, just before the stimulus when the muscle cells are relaxed, and corresponding to the diastolic phase of the contractile cycle in the intact heart, they were stretched to simulate refilling of the heart between beats (Figs. 1*D* and 2*A*). After the stimulus, trabecular length was held constant as active force developed. Sarcomeres shortened during force development (Fig. 2*B*), and continued to shorten for about 100 ms after peak force (PF); SL recovery was slower than force relaxation. The distinct time courses of force and SL indicate the presence of a viscoelasticity or internal load (*SI Appendix*, Fig. S1).

The structural dynamics of the myosin motors and filaments during contraction were determined with nanometer-scale resolution in the same population of sarcomeres by small-angle X-ray diffraction (Fig. 1*C* and Movie S2). The ratio of the intensities of the (1,1) and (1,0) equatorial reflections (*I*_11_/*I*_10_) (Fig. 2*C* and *SI Appendix*, Table S1), commonly used as an index of the movement of myosin motors toward the actin filaments, has the same time course as force (*SI Appendix*, Table S2), consistent with the conclusion from skeletal muscle (18, 19) that force development rapidly follows attachment of myosin motors to actin. The calcium released during contraction of intact electrically paced trabeculae is submaximal; to control the milieu bathing the myofilaments and therefore the available calcium concentration, some of the trabeculae were demembranated, as described in *Materials and Methods*. *I*_11_/*I*_10_ in intact trabeculae in diastole was the same as that in demembranated trabeculae at very low steady calcium concentration [Ca^2+^] (Fig. 2*C*, orange), but *I*_11_/*I*_10_ at PF was lower than that during steady activation at saturating [Ca^2+^] (Fig. 2*C*, blue) and much lower than when all myosin motors are attached to actin in rigor (*SI Appendix*, Table S1), indicating that only a small fraction of motors become attached to actin during contraction of intact trabeculae.

Two X-ray reflections that signal the regulatory state of the myosin filaments (2, 6, 7), *S*_M6_ (Fig. 2*D*) and *I*_ML1_ (Fig. 2*E*) also tracked force development during activation (*SI Appendix*, Table S2), but had distinct time courses during relaxation. *S*_M6_ measures the axial periodicity of the filament backbone, which increases by about 1% during force development (Fig. 2*D*), showing that the backbone of the myosin filament in heart muscle acts as a mechanosensor for filament activation, as in skeletal muscle (2, 6, 7). *S*_M6_ in diastole is close to that at very low [Ca^2+^] (Fig. 2*D*, orange), but *S*_M6_ at PF is lower than at maximal [Ca^2+^] (Fig. 2*D*, blue). Recovery of *S*_M6_ has fast and slow components, with a pause at 220 to 300 ms.

*I*_ML1_, the intensity of the first myosin-based layer-line (Fig. 1*C*), signals the helical packing of the myosin motors on the surface of the filament that inhibits their interaction with actin (2, 7, 20). *I*_ML1_ at PF was about 25% of its diastolic value (Fig. 2*E*). Since diffracted intensities are proportional to the square of the number of diffractors, about half of the motors that are helically ordered in diastole leave that state during force development. Strikingly, *I*_ML1_ did not start to recover until about 300 ms, when force has already returned to about one-third of its peak value.

The results in Fig. 2 define three phases in the relaxation of heart muscle. In phase 1, force decreases as sarcomere shortening continues, but myosin motors detach from actin (*I*_11_/*I*_10_) (Fig. 2*C*) and the axial periodicity of the filament backbone starts to recover (*S*_M6_) (Fig. 2*D*) with no reappearance of the helical arrangement of the motors (*I*_ML1_) (Fig. 2*E*). In phase 2, from about 220 to 300 ms, force continues to decline as sarcomeres reextend, SL dispersion increases (*SI Appendix*, Fig. S1*D*), but there is no change in either filament backbone periodicity or the helical arrangement of the motors. In phase 3, from 300 ms, SL dispersion decreases and the myosin-related structural signals recover to their diastolic levels.

### Only About 10% of the Myosin Motors Are Attached to Actin at PF.

**Fig. 3.**
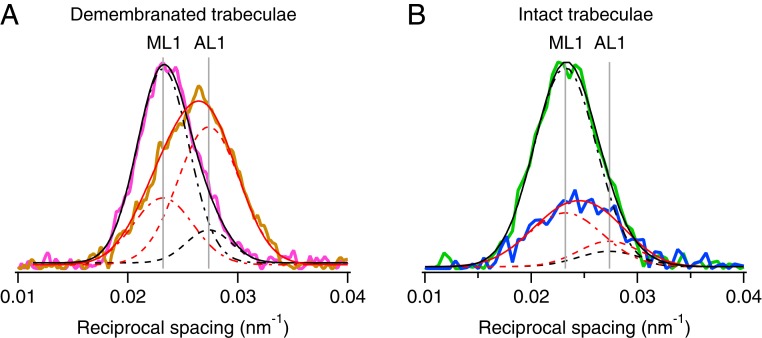
Fraction of myosin motors attached to actin at peak force. (*A*) Axial profiles of the first layer line from demembranated trabeculae in relaxing (pCa 7.0; magenta) and rigor (pCa 9.0, no ATP; orange) conditions. Data added from four trabeculae. FReLoN detector with sample-to-detector distance, 1.6 m. Total exposure time, 160 ms. Temperature, 27 °C. Black and red continuous lines, double-Gaussian global fits to the axial layer-line profiles, with positions of myosin-layer 1 (ML1) and actin-layer 1 (AL1) as common parameters (continuous vertical gray lines). Dot-dashed lines and dashed lines are Gaussian components of the global fits for ML1 and AL1, respectively. (*B*) Axial profiles of the first layer line from intact trabeculae in diastole (green) and at PF (blue). Data added from four time-frames in diastole and around PF. Pilatus detector, sample-to-detector distance, 3.2 m. Total exposure time, 230 ms. Temperature, 26.4 °C. Black and red continuous lines, double-Gaussian fits to the axial profiles with positions of ML1 and AL1 constrained to the vertical gray lines from fits in *A*. Dot-dashed lines and dashed lines as in *A*.

We used the X-ray layer line reflections associated with the axial periodicities of the myosin- and actin-based helices, about 43 and 37 nm, respectively (20), to estimate the number of myosin motors attached to actin at PF. Although the observed axial profiles of these layer lines overlap substantially (Fig. 3), their relative intensities can be determined accurately by Gaussian deconvolution if the spacings of the component layer lines are known. Those component spacings were determined in demembranated trabeculae in relaxing conditions at pCa7 (Fig. 3*A*, magenta), chosen to be close to diastolic conditions in intact trabeculae where the myosin-based helix dominates, and in adenosine triphosphate (ATP)-free or rigor conditions (Fig. 3*A*, orange), where the actin-based helix dominates. Both profiles were well fitted with a first myosin-based layer line spacing (*S*_ML1_) of 43.11 ± 0.04 nm, and a first actin-based layer line spacing (*S*_AL1_) 36.56 ± 0.09 nm (mean ± SD). The same procedure was then applied to the observed layer lines in electrically paced intact trabeculae in diastole (Fig. 3*B*, green) and at PF (Fig. 3*B*, blue). In both cases the profiles were well-fitted by a double Gaussian function with *S*_ML1_ and *S*_AL1_ constrained to the mean values above. The fraction of myosin motors attached to actin (*f*_A_) at PF was estimated from the intensity of the AL1 reflection (*I*_AL1_) using the model of Koubassova et al. (21) under the assumption that all of the motors are attached to actin in rigor. The resulting estimate of *f*_A_ at PF was about 0.10 (see *Materials and Methods* for details), in reasonable agreement with a recent estimate from mechanics (22), 0.08 ± 0.01. Since there are 294 motors in each half thick filament, only about 29 motors are attached to actin at PF.

### About 400 ATP Molecules Are Hydrolyzed per Half-Myosin Filament during Force Development.

**Fig. 4.**
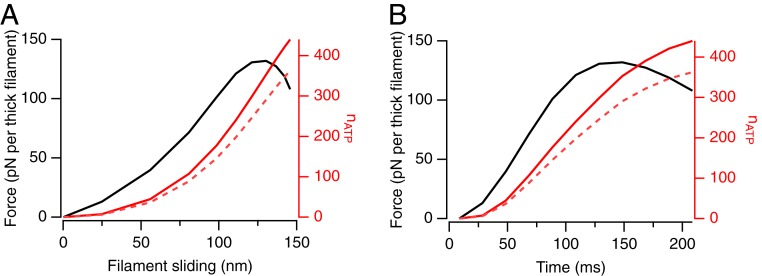
ATP hydrolysis per half-filament during force development and up to peak shortening. Force (black) and the number of ATPs hydrolyzed per half-filament (red), calculated as described in the main text from thermodynamic efficiency (red continuous line) or from the assumption that motors remain attached to actin over a 6-nm stroke (red dashed line), plotted against filament sliding (*A*) or time (*B*).

The conclusion that only about 10% of the motors are attached to actin at PF does not mean that the other 90% are not involved in the process of force development. The number so involved can be estimated from the number of ATP molecules hydrolyzed per half-filament during force development (*n*_ATP_), using the generally accepted assumption that one ATP is hydrolyzed in each cycle of motor interaction with actin. We estimated *n*_ATP_ in the conditions of the present experiments by two independent methods (Fig. 4). First, we estimated the peak force per thick filament as 132 pN from the trabecular force per cross-sectional area (Fig. 2*A*) and *d*_1,0_, the separation of the (1,0) equatorial planes, which is 37.5 nm in diastole in the present experiments, assuming that the myofilament lattice occupies 60% of the cross-sectional area. With 29 motors attached to actin at PF, the force per attached motor is 4.4 pN, close to recent estimates from sarcomere stiffness (22) and single-molecule measurements (23). The work done during force development was then calculated from the filament force and SL time courses (Fig. 2 *A* and *B*) as 9,700 zJ per half-filament. Assuming that the free energy of ATP hydrolysis, ∼100 zJ per molecule, is converted into work with an efficiency of 22% (24), *n*_ATP_ is about 440 at PF (Fig. 4, continuous red line). Alternatively, the work per individual motor cycle can be estimated as 26.4 zJ [4.4 pN over a 6-nm stroke (25)], which leads to an *n*_ATP_ of about 370 at PF (Fig. 4, dashed red line). An independent estimate can be obtained from the total number of motor detachments during force development, calculated from the fraction *n*_A_ of motors attached to actin at each time point (29 at PF and proportionally less at lower forces; see below) and the filament sliding (Fig. 2*B*), assuming that motors can remain attached over filament sliding of 6 nm (25) and that one ATP molecule is hydrolyzed per detachment. This method gives an *n*_ATP_ of about 380 at PF. These estimates suggest that the average ATP hydrolysis per myosin motor is about 400/294 or about 1.3. If only the 10% of motors attached at PF are active during force development, they would each need to hydrolyze about 13 ATPs in about 200 ms, corresponding to a turnover rate per motor of ca 65 s^−1^, an order-of-magnitude greater than values from measurements on the isolated proteins (26). It seems very likely therefore that many more myosins in the half-filament drive force development, but that most of them interact transiently with actin and have detached before PF.

### The PF of Contraction in Electrically Stimulated Heart Muscle Is Borne by Motors in the C-Zone of the Myosin Filament.

**Fig. 5.**
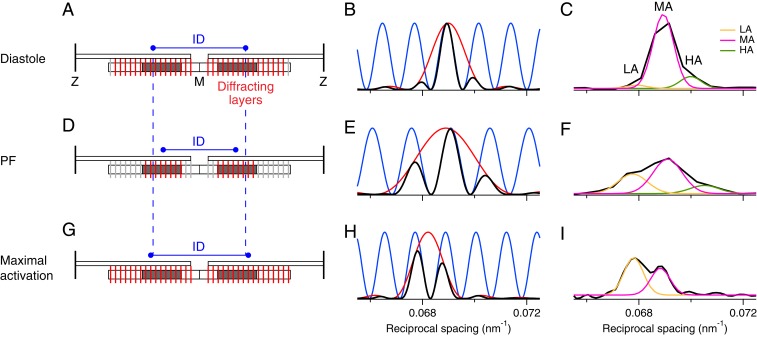
Determining the location of diffracting motors in the myosin filament by X-ray interference. The *Left* column (*A*, *D*, and *G*) shows the (red) ordered layers of myosin motors in each half-thick filament in the sarcomere that contribute to the M3 X-ray reflection and the (light gray) disordered layers that do not. Only one of every three layers of motors is shown for simplicity. The center-to-center distance between the ordered layers is the interference distance (ID, blue). The C-zones are shaded dark gray. The *Center* column (*B*, *E*, and *H*) shows the intensity distribution of the M3 reflection that would be produced by a single array of ordered motors in each half filament (red), the fringes (blue) produced by interference between the two arrays in each filament, and the product of the red and blue functions, the resulting M3 profile (black) for the parameter set specified in *SI Appendix*. The *Right* column (*C*, *F*, and *I*) shows corresponding experimental M3 profiles (black) fitted by multiple Gaussian functions for the lower (LA, orange), middle (MA, magenta), and higher (HA, green) angle peaks. Data in *C* and *F* added from six trabeculae, in *I* from two. The *Top* row (*A*–*C*) is for intact trabeculae in diastole, the *Middle* row (*D*–*F*) for intact trabeculae at peak force, and the *Lower* row (*G*–*I*) for demembranated trabeculae at full calcium activation. Note that the calculated profiles (*Center* column) do not include the broadening due to the point-spread function of the camera/detector system in the experimental profiles (*Right* column), but this difference is avoided in the analysis presented in the text by Gaussian fitting both experimental and calculated profiles.

Next, we used X-ray interference to localize the dynamic structural changes in the myosin motors during contraction to specific domains of the myosin filament. Each half-filament contains 49 layers of myosin motors with an axial spacing of about 14.5 nm, starting about 80 nm from the filament midpoint (M) (Figs. 1*B* and 5*A*). Layers 7 to 31 constitute the myosin binding protein-C (MyBP-C)–containing C-zone (Fig. 1 *A* and *B*, green and Fig. 5*A*, dark gray), 1 to 7 the proximal or P-zone, and 31 to 49 the distal or D-zone (13). X-ray interference between the two arrays of motors in each myosin filament effectively multiplies the axial profile of the M3 X-ray reflection produced by a single array of motors (Fig. 5*B*, red) by a fringe pattern (Fig. 5*B*, blue) with periodicity determined by the distance between the centers of the two motor arrays, the interference distance (ID) (Fig. 5*A*), resulting in a characteristic multipeak profile (Fig. 5*B*, black) (27).

The observed profile of the M3 reflection in diastole (Fig. 5*C*) is consistent with that expected from interference between almost all of the 294 motors in each half-filament (Fig. 5*A*, red), with a dominant central or midangle (MA) peak and small low-angle (LA) and high-angle (HA) satellites, as described previously for resting skeletal muscle (27, 28) and diastolic heart muscle (4). A similar profile was seen in heart muscle at very low [Ca^2+^] (*SI Appendix*, Fig. S2*A*). At PF however (Fig. 5*F*), the LA and HA peaks become both more intense and more widely separated, showing that the M3 profile corresponding to the single motor array has become broader (Fig. 5*E*, red) and that the interference fringes are more widely spaced (Fig. 5*E*, blue), and therefore that the motor array in each half-filament has become shorter and displaced toward the filament midpoint (Fig. 5*D**,* red) (29). In fact the observed relative intensities and positions of the subpeaks of the M3 reflection at PF (Fig. 5*F*) match those expected if the motors contributing to the reflection are confined to the C-zone (Fig. 5*D**,* dark gray). In contrast, at maximal calcium activation, the profile of the M3 reflection becomes narrower once more (Fig. 5 *H* and *I*, red), and the interference fringes are more closely spaced (Fig. 5 *H* and *I*, blue). The observed profile (Fig. 5*I*) is once more that expected from almost all of the 294 motors (Fig. 5*G*, red), as previously reported for full activation of skeletal muscle (27, 28), and at the higher force in trabeculae that are stretched during activation (2).

**Fig. 6.**
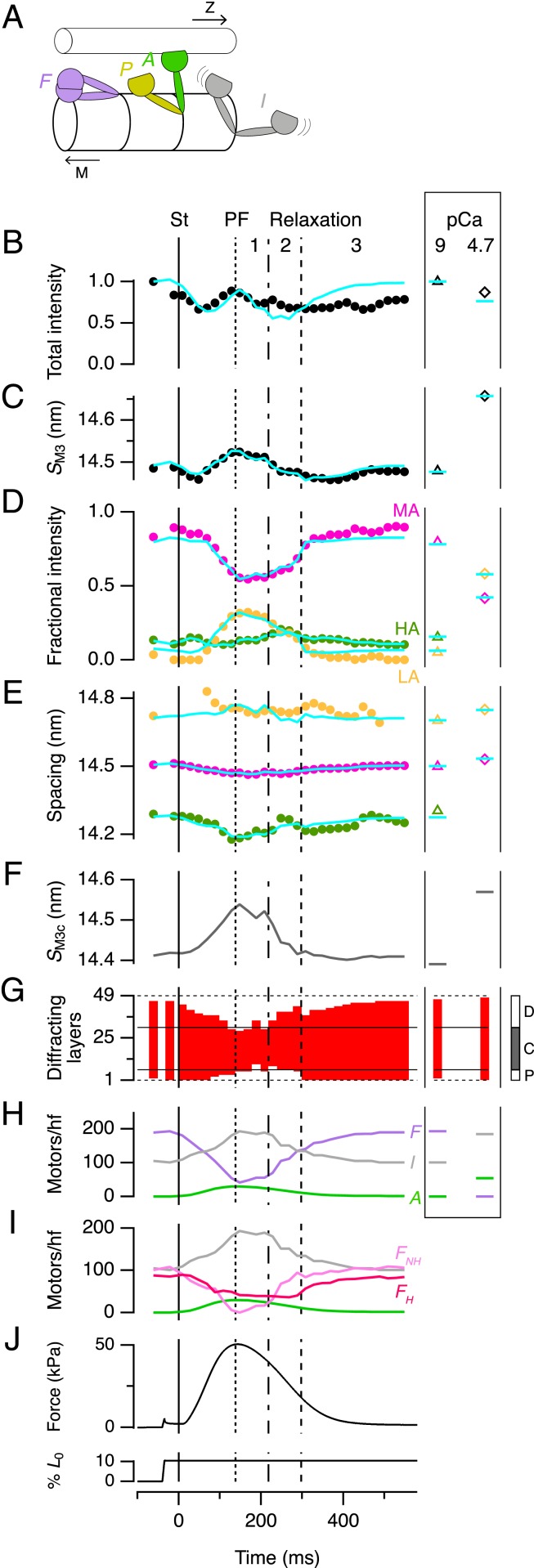
Determining the number of motors in standard conformations by X-ray interference. (*A*) Standard motor conformations: folded (F, purple), actin-attached (A, green), partner of an attached motor (P, yellow), and isotropic (I, gray). Toward filament midpoint, M; Z-band, Z. (*B*–*E*) Time courses of M3 intensity (*I*_M3_, *B*) and spacing (*S*_M3_, *C*), and the fractional intensity (*D*) and spacing (*E*) of its three component peaks with color code as in Fig. 5*C*. Data added from six trabeculae. St, stimulus. Vertical lines as in Fig. 2. (*Right Inset*) Values from demembranated trabeculae in relaxation (triangles) and full activation (diamonds); note that the HA peak cannot be measured at pCa 4.7. Cyan lines denote results from the calculations described in the text for the following parameters: (*F*) Spacing of the myosin motors in the C-zone (*S*_M3c_). (*G*) Diffracting layers of myosin motors (red vertical bars) and their position in the myosin half-filament as shown in the *Inset* at *Right*. (*H*) Number of motors folded, actin-attached and isotropic per half-filament; color code as in *A*. (*I*) The folded motors in *H* resolved into the maximum number in the helical array (*F*_H_, dark pink) and the remainder nonhelical motors (*F*_NH_, light pink). (*J*) Force and trabecular length change reproduced from Fig. 2*A* for reference.

The mean spacing of the M3 reflection (*S*_M3_) was about 1.2% larger during full activation than in diastole (Fig. 6*C*), as can be seen from the leftwards shift of the M3 profile on full activation (Fig. 5 *C* and *I*), similar to that observed on full activation of skeletal muscle (27, 28), but the change in *S*_M3_ at PF in electrically stimulated heart muscle (Figs. 5*F* and 6*C*) is less than 0.25%. In diastole *S*_M3_ is exactly twice *S*_M6_ (*SI Appendix*, Table S1), as expected for a motor periodicity (*S*_M3_) determined by its attachment to the filament backbone, which dominates *S*_M6_. This ratio is retained at full activation in skeletal muscle (27, 28) and, within the precision of the measurements, in heart muscle (*SI Appendix*, Table S1). At PF in heart muscle, however, the *S*_M3_:*S*_M6_ ratio is significantly less than two (2). The structural basis for the uncoupling of *S*_M3_ and *S*_M6_ during contraction of electrically stimulated heart muscle is unknown, but a similar effect occurs transiently on activation of skeletal muscle (18). The results in Fig. 5 suggest a possible explanation. *S*_M6_ measures the backbone periodicity of the whole filament, but *S*_M3_ at PF (but not at full calcium activation) comes only from motors in the C-zone (Fig. 5*D*, red, dark gray), so the observed behavior would be reproduced if the axial periodicity of the myosin filament were about 2% smaller in the C-zone than in the P- and D-zones. The distinct superrepeat of titin, the “molecular ruler” of the myosin filament, in the C-zone (30) makes that hypothesis structurally plausible.

### Numbers of Myosin Motors in Standard Conformations during the Contraction–Relaxation Cycle.

The relative intensities of the interference subpeaks of the M3 reflection at each time point during contraction (Fig. 6*D*) and their spacings (Fig. 6*E*) give precise information about the dynamic conformation of the myosin motors, and the filament location of those motor conformations. Since standard motor conformations have been defined by previous work (2, 25, 28), we used these X-ray interference data to determine the number of motors in each conformation at each time point. We considered four motor conformations: 1) Actin-attached (A) (Fig. 6*A*, green) force-generating motors; 2) “partner” (P) (Fig. 6*A*, yellow) motors in a AP myosin dimer; 3) motors that are folded (F) (Fig. 6*A*, purple) against the filament surface in the “interacting heads motif” (9, 17), superrelaxed (8) or “OFF state” (6, 7) that makes them unavailable for actin-interaction; and 4) isotropic (I) (Fig. 6*A*, gray) or disordered motors, which would not contribute to the M3 reflection. The number of A motors per half-filament at PF (*n*_A_) is about 29, as discussed above; at other times *n*_A_ is likely to be proportional to force, as indicated by the time course of the equatorial X-ray reflections (Fig. 2*C*), and by mechanical and X-ray data from skeletal muscle (18, 28). *n*_P_ = *n*_A_ by definition, so the only parameters required to calculate the axial profile of the M3 reflection are the number of folded motors (*n*_F_), the axial periodicity of motors (*S*_M3_), assumed to be 2% shorter in the C-zone as discussed above, and the first and last layer of the array of ordered motors in each half-filament (Fig. 5 *A*, *D*, and *G*, red), assumed for simplicity to be contiguous.

We used a global search of these four parameters (*SI Appendix*) to determine the best-fit to the relative intensities (Fig. 6*D**,* cyan lines) and absolute spacings (Fig. 6*E*, cyan lines) of the LA, MA, and HA components of the M3 reflection at each time point during contraction of electrically paced intact trabeculae (Fig. 6, circles) and in demembranated trabeculae in relaxation (Fig. 6, box at right, triangles) and full activation (Fig. 6, diamonds). The global best-fit parameters reproduced the relative intensities and spacings of the subpeaks of the M3 reflection (Fig. 6 *D* and *E*), *S*_M3_ (Fig. 6*C*) and, to a first approximation, the total intensity (*I*_M3_) (Fig. 6*B*) at each time point and in each condition. The axial periodicity of the C-zone, *S*_M3c_ (Fig. 6*F*), increased by about 0.9% at PF, similar to the increase in *S*_M6_ (Fig. 2*D*), supporting the working hypothesis from the previous section that motors follow the backbone periodicity of the myosin filament during contraction, but the filament periodicity is 2% shorter in the C-zone.

**Fig. 7.**
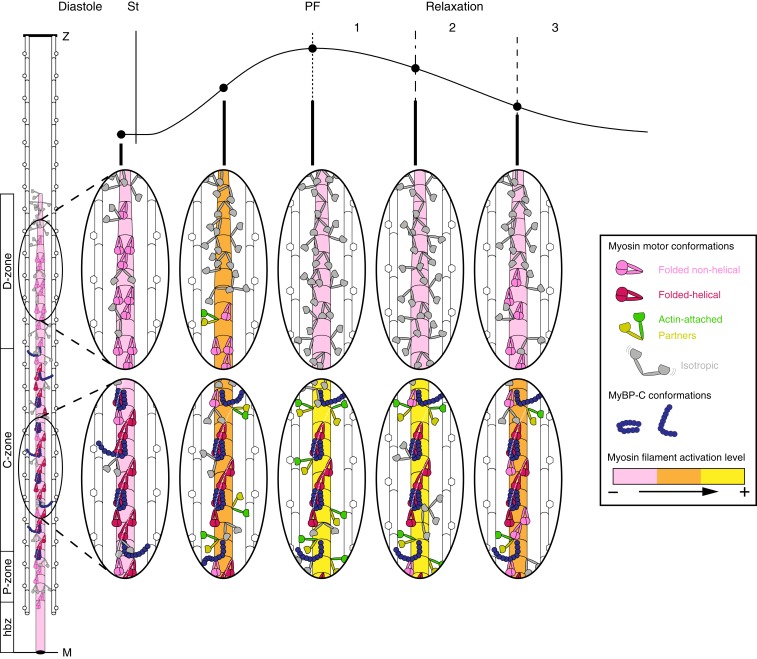
Myosin motor conformations and regulatory states of myosin filament zones during contraction. (*Left*) Motor conformations in the half-sarcomere in diastole: Folded and helically ordered (*F*_H_, dark pink), folded nonhelical (*F*_NH_, light pink), and isotropic (*I*, gray). MyBP-C (blue) may link to actin filaments or be associated with helical motors. (*Right*) Magnification of C- and D-zones at the times indicated by the circles on the force trace. St, stimulus. Actin-attached motors (A, green); partners of A motors (P, yellow). Regulatory state of the myosin filament backbone indicated as pink (more off) through orange to bright yellow (more on).

The results of these calculations confirm and quantify the conclusions from Fig. 5. In diastole almost all of the motor layers in each half-filament are ordered (Fig. 6*G*). The number of ordered layers decreases during force development, and at PF only layers 8 ± 3 to 32 ± 2 (mean ± SD) are ordered (Fig. 6*G*), consistent with the boundaries of the C-zone determined by electron microscopy (13) and immunofluorescence (Fig. 1*A*), which is from layers 7 to 31 (Fig. 6 *G*, *Right Inset*, gray). Since A motors are highly ordered (25), we conclude that the peak force of contraction is borne by A motors in the C-zone. *n*_F_ at PF was 41 ± 14 (Fig. 6*H*). Since the other layers are disordered, these folded motors must also be in the C-zone. Helical motors are folded, so the number of helical motors at PF cannot be more than 41 ± 14. The change in the intensity of the ML1 layer line (Fig. 2*E*) shows that the number of helical motors at diastole is about twice that at PF, so the maximum number of helically ordered motors in diastole cannot be more than 82 ± 28. This number would be completely accounted for by about two-thirds of all of the motors in the C-zone (17), making it very unlikely that the ∼105 folded motors in the P- and D-zones in diastole (Figs. 6*I* and 7, light pink) are helically ordered. This result shows that there are both folded helical (Figs. 6*I* and 7, dark pink) and folded nonhelical motors (Figs. 6*I* and 7, light pink), and that the folded helical motors are confined to the C-zone.

### Length-Dependent Activation and the Frank–Starling Mechanism.

Stretching trabeculae in diastole increases the active force in the next contraction (Fig. 1*D*). This response, called length-dependent activation (LDA), is physiologically important as the cellular basis of the stronger heartbeat that follows increased return of venous blood in the intact heart, the Frank–Starling mechanism (31). Some previous X-ray studies have suggested that LDA is mediated by stretch-activation of the myosin filament in diastole (32), while others (33) reached the opposite conclusion. Here, as in ref. 32, we applied the stretch just before a stimulus to isolate the immediate effect of the length change from slower changes in intracellular calcium handling but, like ref. 33, we limited the amplitude of stretch to minimize the change in passive force, while maintaining a robust LDA response, almost doubling the active force in response to the next stimulus (Fig. 1*D*). We found that diastolic low-force stretch can produce a large LDA response without significant change in the regulatory state of the myosin filament as monitored by either *S*_M6_ or *I*_ML1_ (Fig. 2 *D* and *E*), in agreement with the conclusions of ref. 33. Moreover diastolic stretch produced no change in the conformation of the myosin motors as determined from the interference fine structure of the M3 X-ray reflection and its interpretation in terms of motor populations (Fig. 6). These results suggest that the Frank–Starling response of the intact heart is not mediated by an immediate change in myosin filament structure accompanying the increase in myosin filament stress on diastolic stretch, and that an additional mechanism of length-sensing must underlie LDA. That additional mechanism remains to be elucidated, but seems to involve the actin as well as the myosin filament (31).

### Control of the Dynamics of Force Development.

The numbers of motors in each conformation at each time point during contraction (Fig. 6*I*), combined with the earlier conclusion that about 400 motors per half filament hydrolyze ATP during force development (Fig. 4), but only ∼29 motors in the C-zone bear the active force (Figs. 5 and 6), lead to a novel description of contractile dynamics in heart muscle (Fig. 7). During force development, helical and folded motors are lost with about the same half-time as the rise of force (Fig. 6*I* and *SI Appendix*, Table S2), with *S*_M6_ slightly faster, consistent with the mechanosensing hypothesis of filament activation described for skeletal muscle (6, 7). That hypothesis postulated that some motors are constitutively ON (i.e., they are not in the folded OFF state) even at low levels of actin filament activation; the present results show that these constitutively ON motors are likely to be in the D- (or P-) zone (Fig. 7, diastole). D-zone motors are closer to the sites of calcium release and therefore the first to be activated (16), which would stress-activate the remainder of the filament according to the mechanosensing paradigm (2, 7). D-zone motors are also nonhelical and not subject to inhibition by MyBP-C (14). During force development, D-zone motors become more disordered (Figs. 6*G* and 7); at PF they are all isotropic and, because there are no actin-attached motors in the D-zone, there is no stress in the D-zone filament backbone, inhibiting the D-zone by mechanosensing (Fig. 7, PF). In contrast, actin filaments in the C-zone are sensitized to calcium by binding of the N terminus of MyBP-C (14, 15), and remain active for longer. The dual effect of MyBP-C to inhibit myosin filaments but activate actin filaments (14) can explain both the earlier activation and earlier inactivation of the D-zone relative to the C-zone.

### Control of the Dynamics of Relaxation.

Mechanical relaxation, in contrast to force development, is primarily determined by detachment of the C-zone motors, suggesting a possible explanation for the key role of the phosphorylation state of MyBP-C in the dynamics of relaxation (34). The structural and zonal dynamics of relaxation have three kinetic phases (Figs. 2 and 7). In phase 1, from PF to peak shortening at about 220 ms, a few motors detach from actin as signaled by the decrease in force and *I*_11_/*I*_10_ (Fig. 2*C*). Filament backbone strain decreases roughly in proportion to force (*S*_M6_) (Fig. 2*D*), but there is almost no recovery of the axial periodicity of the C-zone (*S*_M3c_) (Fig. 6*F*), indicating that the C-zone remains on, presumably still stabilized by MyBP-C binding to the actin filaments, and that the fast phase of the recovery of filament backbone strain (*S*_M6_) (Fig. 2*D*) is due to switching off of the D-zone. In phase 2, from about 220 to 300 ms, force continues to decline as sarcomeres reextend, SL dispersion increases (*SI Appendix*, Fig. S1*D*), and F motors reappear (Fig. 6*H*). *S*_M3c_ recovers (Fig. 6*F*), signaling switching off of the C-zone, but remarkably there is no further recovery of either the filament backbone periodicity (*S*_M6_) (Fig. 2*D*) or the helical arrangement of the motors (*I*_ML1_) (Figs. 2*E* and 6*I*), showing that the ordered motors that appear in the D-zone in phase 2 are folded but not helical (Figs. 6*I* and 7). In phase 3, from 300 ms, SL dispersion decreases, P-zone motors become ordered, the diastolic motor helix is reformed in the C-zone (Fig. 6*I*), and the recovery of the filament backbone periodicity is completed (Fig. 2*D*).

These structural dynamics show that the myosin filament switches on and off in the zonal sequence D to C to P, as expected from the mechanosensing hypothesis of filament activation, since filament stress decreases from the M-band to the filament tip. In addition, folding of myosin motors against the filament backbone is kinetically dissociated from reformation of the helical arrangement of the motors described previously for isolated filaments (9, 17). Folding may be stabilized by an intramolecular motor–tail interaction, whereas the helical state may require additional intermolecular interactions that are coupled to the shorter periodicity of the filament backbone, linked to the presence of MyBP-C or the distinct titin periodicity in the C-zone.

The results described above establish a paradigm for myosin-based regulation of the strength and time course of contraction in heart muscle in which myosin motors in the different domains of the filament play distinct and previously unrecognized roles. The detailed relationship between these filament-level mechanisms and the upstream signaling pathways remains to be determined, and extended to the physiological cardiac cycle and to disease models. The results presented here establish the conceptual and experimental basis for that task.

## Materials and Methods

### Preparation of Cardiac Trabeculae and Experimental Protocol.

*Rattus norvegicus*, strain Wistar Han (male, 6- to 8-wk-old) were supplied by Charles River Laboratories and hosted at the animal house of the home institution (King’s College London) or of the Bio-Medical Facility of the European Synchrotron Radiation Facility (ESRF) at 20 °C, 55% relative humidity, and 12-h light/dark cycles. Food and water were provided ad libitum. On the day of the experiment, the rats were culled by cervical dislocation after sedation with isoflurane in compliance with the UK Home Office Schedule 1 and European Union regulation (directive 2010/63), followed by a confirmation method. The heart was rapidly excised and cannulated via the ascending aorta and retrogradely perfused with a modified Krebs-Henseleit buffer (119 mM NaCl, 5 mM KCl, 0.5 mM CaCl_2_, 1.2 mM NaH_2_PO_4_, 1.2 mM MgSO_4_, 25 mM NaHCO_3_, 10 mM glucose, 25 mM BDM) equilibrated with carbogen (95% O_2_, 5% CO_2_). Single unbranched trabeculae were dissected from the right ventricle under a stereomicroscope and “scorpion-like” clips made of stainless-steel wire were mounted on the valve and ventricular wall ends of each trabecula. Trabecular length (*L*_0_) and cross-sectional area were measured after stretching to just above slack length. They were then mounted in the experimental trough filled with the same buffer between the levers of a strain gauge force transducer and a motor (322C, Aurora Scientific Inc.) at slack length. The trabecula was then perfused with a buffer containing 1.4 mM CaCl_2_ without BDM at 26.4 ± 0.2 °C (mean ± SD, *n* = 6 trabeculae). Two mica windows carrying platinum stimulating electrodes were positioned as close as possible to the trabecula to minimize the X-ray path in solution, and the trabeculae were electrically paced at 1 Hz for at least 30 min before the start of the experiment. SL was measured at multiple points along the trabecula by ultrasmall angle X-ray diffraction at 31-m sample-to-detector distance, and the average SL in diastole was set to 1.94 ± 0.03 μm. Once per minute a ramp stretch (10% *L*_0_ in 5 ms) was applied in diastole 40 ms before the stimulus (*SI Appendix*, Fig. S1) followed by a release to the original length after mechanical relaxation, 600 ms after the stimulus. This transient ramp stretch-release protocol allowed us to average data from repeated activations at the longer SL without altering the calcium transient (31).

Isolated intact trabeculae were fixed and stained with a monoclonal mouse antisarcomeric α-actinin antibody (clone EA-53, Sigma-Aldrich) and a polyclonal rabbit anti–MyBP-C antibody, kindly donated by M. Gautel, King’s College London, London, UK; for details on the protocol, see Iskratsch et al. (35). The confocal micrograph (Fig. 1*A*) was collected at room temperature on a LEICA SP5 confocal microscope equipped with argon and helium-neon lasers and 63× oil immersion objective in sequential scanning mode.

For experiments on demembranated trabeculae, isolated trabeculae were demembranated for 20 min on ice in relaxing solution in the presence of BDM (25 mM) and Triton X-100 1% (vol/vol), then stored at −20 °C in storage solution (6 mM Imidazole, 70 mM KPr, 8 mM MgAc_2_, 5 mM EGTA, 7 mM Na_2_ATP, 1 mM NaN_3_, 50% glycerol) for up to 24 h. Aluminum T-clips were attached to the ends of the trabeculae and they were mounted in a temperature-controlled multidrop apparatus (36) in relaxing solution at ∼2.15-µm SL between the levers of a strain gauge force transducer and the Aurora motor. Before each experiment, the ends of the trabeculae were fixed with shellac dissolved in ethanol at 2 °C. Relaxing solution contained: 25 mM Imidazole, 45 mM KPr, 6.89 mM MgAc_2_, 10 mM EGTA, 5.56 mM Na_2_ATP, 20 mM Na_2_-creatine phosphate (CP), (pCa= −log [Ca^2+^] = 9). Preactivating solution contained: 25 mM Imidazole, 46 mM KPr, 6.48 mM MgAc_2_, 0.1 mM EGTA, 9.9 mM HDTA, 5.6 mM Na_2_ATP, 20 mM Na_2_CP (pCa 9). Activating solution contained: 25 mM Imidazole, 46 mM KPr, 6.39 mM MgAc_2_, 10 mM CaEGTA, 5.65 mM Na_2_ATP, 20 mM Na_2_CP (pCa 4.7). Rigor solution contained: 25 mM Imidazole, 134 mM KPr, 1.5 mM MgAc_2_, 10 mM EGTA (pCa 9). A solution matching the physiological calcium concentration in diastole (pCa 7) was obtained by mixing 62.5% and 37.5% of relaxing and activating solution (vol/vol), respectively. In all of the solutions free [Mg^2+^] = 1.0 mM, ionic strength = 180 mM and pH 7.1 at 25 °C. The osmotic agent dextran T500 (3% [wt/vol]) was added to all experimental solutions (except rigor) to reduce the interfilament spacing to a value similar to that of intact trabeculae (37). Just before the experiment, Protease inhibitor mixture P8340 (Sigma) and 2 mM DTT were added to all of the solutions. The trabecula was activated with a temperature-jump protocol: it was initially equilibrated in preactivating solution at 2 °C for 5 min, then transferred to 1) activating solution at 2 °C for ∼8 s to reach a steady force, 2) activating solution at 27 °C, 3) in air for the X-ray exposure, and 4) finally back to relaxing solution.

### X-ray Data Collection.

For intact trabeculae, the trough was sealed to prevent solution leakage, and the trabecula was mounted vertically at beamline ID02 of the ESRF, which provided up to 2 × 10^13^ photons s^−1^ at 0.1-nm wavelength in a beam of size 300 μm (horizontal, full width at half-maximum) and 70 μm (vertical) at the sample position (38). The beam was attenuated to 3% (25-μm Fe attenuator) for trabecula alignment. To minimize radiation damage, X-ray exposure was limited to the data collection period using a fast electromagnetic shutter (nmLaser Products) and the trabecula was moved vertically by 100 to 200 μm between the contractions used for X-ray data acquisition. X-ray data were collected from the central region (1.1 ± 0.2 mm, mean ± SD) of the trabecula, avoiding the less uniform or weakly diffracting regions near the attachments and the higher collagen content near the valve end. Maximum full-beam exposure time at each position was ∼20 ms. Data were collected from 12 to 30 contractions in each trabecula; peak systolic force (*T*_PF_) decreased by less than 10% during the series and there was no detectable sign of radiation damage in the X-ray diffraction pattern. X-ray data were recorded using the photon-counting detector Pilatus 300K (Dectris), composed of three modules, total area 83.8 mm × 106.5 mm with 487 × 619 pixels, pixel size 172 μm × 172 μm. Seventeen-pixel gaps between the modules split the meridional pattern into three; equatorial and the myosin-based ML1/M1 and M2 reflections were in the middle module; the M3 and M6 reflections were near the edges of the outer modules (Movie S2). The sample-to-detector distance was set to either 31 m to record the sarcomeric reflections or 3.2 m to record the meridional reflections up to the M6. In each activation 37 consecutive frames were recorded from a local region of the trabecula at 20-ms intervals; in each frame data were collected for 17 ms followed by 3-ms dead time. The main advantage of this protocol and detector over previous experiments combining a fast shutter with a slow read-out charge-coupled device (CCD) detector is that every time point comes from the same combination of trabecular regions; its main limitation is that each point on the trabeculae is exposed for 740 ms, which would produce radiation damage if the full beam intensity were used. All time-resolved experiments therefore used a 25-μm Fe attenuator to reduce beam intensity to 3%, so that equivalent full-beam exposure was 22.2 ms (= 740 ms × 0.03). Signal-to-noise ratio was increased by signal-averaging 12 to 30 contractions per trabecula for six trabeculae with length *L*_0_ = 2.5 ± 0.5 mm, cross-sectional area = 59,000 ± 26,000 μm^2^ and PF *T*_PF_ = 50.4 ± 10.0 kPa (mean ± SD).

For X-ray experiments on demembranated trabeculae, a multidrop apparatus provided rapid solution exchange of vertically mounted trabeculae at the beamline. X-ray diffraction patterns were recorded using the high spatial resolution FReLoN CCD detector (38), with active area 50 mm × 50 mm, 2,048 × 2,048 pixels, pixel size 24 μm × 24 μm. X-ray patterns were binned by 8 in the horizontal direction before readout to increase the signal-to-noise ratio along the meridional axis. Data from two to four trabeculae were added for Figs. 2 *C* and *D*, 3, 5*I*, and 6 *B*–*E* and *SI Appendix*, Fig. S2 and Table S1 to further increase the signal-to-noise ratio. Average cross-sectional area was 37,000 ± 11,500 μm^2^ (mean ± SD) and length 1.55 ± 0.35 mm.

### X-ray Data Analysis.

Small angle X-ray diffraction data were analyzed using the SAXS package (P. Boesecke, ESRF, Grenoble, France), Fit2D (A. Hammersley, ESRF, Grenoble, France), and IgorPro (WaveMetrix, Inc.).

#### Analysis of the ultrasmall angle X-ray diffraction patterns collected at 31-m sample-to-detector distance.

The meridional intensity distribution was obtained from the two-dimensional (2D) X-ray patterns recorded at two to four locations in the central region of each trabecula by integrating from 0.39 μm^−1^ on either side of the meridional axis (parallel to the fiber axis). The intensity, spacing, and axial width of the second order of the sarcomere repeat were determined by fitting a Gaussian peak in the region 0.74 to 1.24 μm^−1^. SL dispersion was estimated from the axial width of the reflection after deconvolution of the axial width of the X-ray beam at the detector.

##### Analysis of the small-angle X-ray patterns collected at 3.2-m sample-to-detector distance.

For each trabecula the time series of 2D patterns from each contraction were added, centered, and aligned using the equatorial 1,0 reflections, then mirrored horizontally and vertically. The equatorial intensity distribution was determined by integrating from 0.0045 nm^−1^ on either side of the equatorial axis (perpendicular to the trabecular axis), and the intensities and spacings of the 1,0 and 1,1 reflections were determined by fitting two Gaussian peaks in the region 0.019 to 0.064 nm^−1^ with the constraint *d*_11_ = *d*_10_/(3)^1/2^. Equatorial data with adequate signal-to-noise could be obtained from single trabeculae. Analysis of the meridional and layer line reflections required data averaging from six trabeculae and 1:2:1 smoothing of the 20-ms time frames. The distribution of diffracted intensity along the meridional axis of the X-ray pattern (parallel to the fiber axis) was calculated by integrating from 0.009 nm^−1^ on either side of the meridian. The first myosin and first actin layer lines (ML1 and AL1) were integrated radially in the region between 0.063 and 0.036 nm^−1^ from the meridional axis. The experiments were completed in two visits to the ESRF. Data from the two different visits had slightly different values of energy; therefore the 2D X-ray diffraction patterns could not be added directly, but meridional and layer line intensity distributions from the three trabeculae acquired in each visit were calculated as a function of reciprocal spacing using the *S*_M3_ calibration described below before adding. Background intensity distributions were fitted using a convex hull algorithm and subtracted; the small residual background was removed using the intensity distribution from a nearby region of the pattern containing no reflections. Integrated intensities were obtained from the following axial regions: M3, 0.066 to 0.072 nm^−1^; M6, 0.134 to 0.140 nm^−1^; ML1, 0.017 to 0.024 nm^−1^. The axial limits for ML1 shown in Fig. 2*E* were chosen to exclude the contribution of the first actin layer line. The cross-meridional width of the M3 reflection was determined from the radial distribution of its intensity in the axial region defined above using a double-Gaussian function centered on the meridian, with the wider component considered to be background. The interference components of the M3 reflection were determined by fitting multiple Gaussian peaks with the same width to the meridional intensity distribution. The total intensity of the reflection (*I*_M3_) was calculated as the sum of the component peaks, and the spacing of the reflection (*S*_M3_) as the weighted average of the axial spacing of the component peaks. The combined instrumental point spread function was negligible compared with the radial width of the M3 reflection. The meridional intensity in the region of the M6 reflection was fitted with one or two Gaussian peaks with the same axial width, and its spacing (*S*_M6_) was calculated as the weighted average of the axial spacing of the component peaks. Force, stimulus, fiber length change, and X-ray acquisition timing were collected and analyzed using custom-made software written in LabVIEW (National Instruments).

For experiments on demembranated trabeculae, data at 31-m and 1.6-m sample-to-detector distance were collected using the high spatial-resolution FReLoN CCD detector. Equatorial intensity distributions could be analyzed in single trabecula, while to obtain enough S/N on myosin-based reflections data from two to four trabeculae were added before the analysis as described above.

For the more complete analysis of the mixed layer line reported in Fig. 3, the axial profile of the observed layer line was fitted in the axial region 0.01 to 0.045 nm^−1^. To compare reflection intensities of intact and demembranated trabeculae, we normalized *I*_AL1_ from each preparation by *I*_ML1_ in the same preparation in diastolic and relaxed pCa 7 conditions, respectively, under the assumption that myosin filament structure is the same in those two states. Because there is a significant AL1 in both relaxed and diastolic conditions, its intensity in those two conditions was subtracted from that in rigor and around peak force, respectively, in order to determine the ratio peak force:rigor for the AL1 intensity component associated with strongly attached myosin motors. There is some uncertainty about the application of the relationship between *f*_A,PF_ and *I*_AL1_ relative to rigor in the model of Koubassova et al. (figure 6A in ref. 21) because their value of *I*_AL1_ in the absence of strongly bound motors is much less than that observed in trabeculae in diastole, and the relationship has a significantly lower slope at the lower extreme of *I*_AL1_. Depending on the interpretation of the diastolic AL1, the resulting estimate of *f*_A_ at peak force is about 0.10. Data for diastole were added from four time-frames (at −21.5, 8.5, 548.5, and 578.5 ms from the stimulus) and for peak force from 108.5 to 168.5 ms from the stimulus.

### Calibration of Spatial Periodicities.

Two-dimensional X-ray diffraction patterns were acquired from intact quiescent and relaxed demembranated rat cardiac trabeculae, and from relaxed demembranated skeletal fibers from rabbit psoas muscle using the high spatial-resolution FReLoN CCD detector at sample-to-detector distances 1.6 m, 2.4 m, and 3.2 m. The psoas muscle fibers were prepared as previously described (39) and mounted in relaxing solution containing 5% dextran T500 (wt/vol), temperature, 27 °C. At each sample-to-detector distance, 2D-patterns were centered and aligned using the equatorial 1,0 reflections, and the distribution of diffracted intensity along the meridional axis of the X-ray pattern was calculated by integrating from 0.005 nm^−1^ on either side of the meridian. The interference components of the M3 reflection were determined by fitting multiple Gaussian peaks with the same axial width to the meridional intensity distribution. The spacing of the reflection was calculated in pixels from the center of the pattern (*S*_M3,pixel_) as the intensity-weighted average of the spacings of the component peaks. The relationship between S_M3,pixel_ and sample-to-detector distance (*L*) for the three values of *L* was fitted by linear regression and the scattering angle θ was determined from the arctangent of its slope. The spacing of the M3 reflection in nanometers was then calculated as *S*_M3_ = λ/(2sin(θ/2)), where λ was estimated from the monochromatic beam energy calibrated with Au L_3_ and Pb L_3_ edges. *S*_M3_ in intact quiescent trabeculae was 14.479 ± 0.007 nm; *n* =1; SL = 1.95 μm; in relaxed demembranated trabeculae 14.479 ± 0.011 nm; *n* =2; SL = 2.14 ± 0.04 μm, and in relaxed skeletal muscle fibers 14.461 ± 0.013 nm; *n* =3; SL = 2.47 ± 0.05 μm. These values are not significantly different from each other (*P* = 0.08, two-tailed *t* test), but are ∼1% larger than those previously reported (40).

### Data Availability.

All relevant data, associated protocols, and materials are within the paper and *SI Appendix*. If any additional information is needed, it will be available upon request from the corresponding author.

## Supplementary Material

Supplementary File

Supplementary File

Supplementary File
