# Supplementary material for: Myosin filament-based regulation of the dynamics of contraction in heart muscle
Source: Proc Natl Acad Sci U S A. 2020 Mar 27;117(14):8177–86. doi: 10.1073/pnas.1920632117 (PMC7149498; doi:10.1073/pnas.1920632117)
Supplement: Supplementary File [file pnas.1920632117.sapp.pdf]

PNAS
www.pnas.org

Supplementary Information for

Myosin filament-based regulation of the dynamics of contraction in heart muscle.

Elisabetta Brunello[a,1], Luca Fusi[a], Andrea Ghisleni[a], So-Jin Park-Holohan[a], Jesus Garcia Ovejero[a], Theyencheri Narayanan[b], Malcolm Irving[a]

[a]Randall Centre for Cell and Molecular Biophysics, School of Basic and Medical Biosciences and BHF Centre of Research Excellence, King's College London, London SE1 1UL, UK. [b]European Synchrotron Radiation Facility, Grenoble 38043, France.

[1]Correspondence to: Elisabetta Brunello, elisabetta.brunello@kcl.ac.uk

This PDF file includes:

Supplementary text
Figures S1 to S3
Tables S1 to S2
Legends for Movies S1 to S2
SI References

Other supplementary materials for this manuscript include the following:

Movies S1 to S2

www.pnas.org/cgi/doi/10.1073/pnas.1920632117

Supplementary Information Text

Interpretation of the profile of the M3 reflection in terms of myosin motor conformations. Myosin filaments from vertebrate skeletal and cardiac muscle have a bipolar structure that is symmetrical about their midpoint at the M-band of the sarcomere (Fig. 1*B*). Each half-filament has 49 layers of myosin motors with a periodic repeat of about 14.5 nm, corresponding to the spacing of the M3 X-ray reflection (S_{M3}), with the first layer (layer 1) starting about 80nm (the half bare zone, *hbz*) from the filament midpoint. The C-zone of the myosin filament, containing myosin binding protein-C (MyBP-C) extends from layers 7 to 31 in heart muscle (1); layers 1 to 7 are denoted the proximal or P-zone, and layers 31 to 49 the distal or D-zone (Fig. 1B).

Each of the 49 layers contains 3 dimeric myosin molecules, or 6 motor domains. Each motor domain is considered to be in one of four possible conformations defined by previous studies (2-5). The model is constrained by the one-dimensional projection of the motor mass distribution onto the filament axis, as inferred from the axial or meridional part of the X-ray diffraction diagram. The available information about the axial mass projection can be expressed in terms of two parameters. Δz is the center of mass of the distribution with respect to the junction between the motor and its adjoining rod domain, the point of attachment to the filament backbone, defined as positive for displacements away from the filament mid-point. The other parameter is usually expressed in terms of the width of a Gaussian distribution, either of motor mass or of the orientation of its lever arm (2-4, 6). Here we use a simpler approach, representing each motor conformation as a point mass at Δz with a weight w that represents the inverse width of the mass distribution; narrower distributions have a larger w and a greater contribution to diffracted intensity. The point-diffractor representation is an excellent approximation for the purpose of calculating the profile of the M3 reflection, since the crystallographic form factor corresponding to the Fourier transform of the axial mass distribution is effectively constant over that profile (7).

In the formalism described above the four motor conformations (Fig. 6*A*) are defined as follows:

- *F*: motors that are *folded* back against the filament surface in the 'interacting heads motif' (8, 9) or OFF state (10, 11) that makes them unavailable for interaction with actin. Δz_F is taken as -7.97 nm (2, 3) and the *F* state is taken as the reference structure so w_F = 1.
- *A*: *actin-attached* or force-generating motors, with Δz_A = +3.03 nm ('model 1' of ref. 2) and w_A = 2.19. Δz_A was determined from the results of length- or load-step X-ray experiments in skeletal muscle (4, 5, 12, 13) as described previously (2, 3). w_A was calculated from the width of the mass distributions of *A* motors determined from those experiments in comparison to the reference weight for the *F* conformation (3). For simplicity Δz_A and w_A were assumed to be independent of mechanical conditions or time.
- *P*: Partner (*P*) (2, 6) or 'active detached' (3) motors, also originally characterized by length- and load-step X-ray experiments in skeletal muscle, with Δz_P = +2.07 nm and w_P = 2.19. The designation 'partner' reflects the evidence from skeletal muscle that these motors constitute part of a myosin dimer with the *A* motors during isometric contraction, and imposes the constraint that the number of *P* motors is equal to that of *A* motors.
- *I*: isotropic or disordered motors that do not contribute to the M3 reflection, either because their range of conformations is so large that their mass is evenly spread over the 14.5-nm axial repeat of the motor layers, or because they have lost their registration on the myosin filament backbone. Thus w_I = 0.

The above formalism was applied by considering the myosin filament as a 1D lattice of point diffractors with mirror symmetry about the filament midpoint, with the first lattice point in each half-filament at the half bare zone (z_1 = *hbz*), followed by the other 48 layers. *hbz* was 87.72 nm in diastole, and was assumed to increase with increased filament stress as expected for the measured myosin filament compliance in skeletal muscle (3). The diffractor at layer *m* has axial periodicity S_{M3m}, and weight *w* and displacement Δz corresponding to its conformation. The

contribution to the amplitude of the M3 reflection from a given conformation was calculated from eq. 1:

$$G = \sum_{m=m1}^{m=m2} \cos\left(2\pi R \cdot (z_m + \Delta z)\right) \qquad \text{eq. 1.}$$

where R is the reciprocal space co-ordinate, z_m the distance of the head-rod junction of the *m*-layer of motors from the filament midpoint, and *m1* and *m2* represent the first and last layers in a contiguous array of point diffractors in each half-filament, layers outside that region making zero contribution because their motors are in the *I* conformation. Multiple conformations (*i*) were introduced with weight w_i defined above and number of motors n_i, to calculate the intensity of the M3 reflection as a function of the reciprocal spacing R as in eq.2:

$$I(R) = \left(\sum_i G_i \cdot w_i \cdot n_i\right)^2 \qquad \text{eq. 2.}$$

$n_A = n_P$ was assumed to be zero in diastole and 29 at peak force, corresponding to 10% of the total number of motors in the myosin half-filament (Fig. 3); the values at other times were calculated under the assumption that n_A is proportional to the force at each time point. Similar results were obtained with n_A at peak force in the range 23 to 35 suggested by previous studies (2, 14). *A*, *P* and *F* motors were assumed to be confined to the ordered layers, and equally distributed within those layers.

The axial profile of the M3 reflection ($I(R)$) was calculated for each set of values of the four adjustable parameters n_F, S_{M3m}, *m1* and *m2* and fitted with three Gaussian distributions of the same width, using the same fitting algorithm as for the experimental data (Fig. 5*C,F,I*), giving the calculated relative intensities (Fig. 6*D,* cyan line) and absolute spacings (Fig. 6*E,* cyan line) of the LA, MA and HA components of the M3 reflection. This approach provides a precise comparison with the corresponding experimental parameters (circles) without the additional step of convolution of the point-spread functions of the X-ray beam and detector, which were themselves well-fitted by Gaussian functions. The axial profiles in Fig. 5 *B,E,H* were obtained using the following parameters respectively: *hbz*, 80.1, 86.1, 83.7 nm; S_{M3}, 14.495, 14.515, 14.66 nm; *m*1, 2, 6, 1; *m*2, 45, 30, 48. The interference distance *ID* in that Figure was calculated as $2{*}hbz+(m1+m2-2){*}S_{M3}$. For Fig. 6, the best fit to the relative intensities and spacings of the LA, MA and HA components (constituting five independent parameters since the total intensity I_{M3} was not used as explained below) at each time point was determined by a global search of n_F, S_{M3m}, *m1* and *m2*, by minimising χ^2 calculated using the experimental SDs from the multiple Gaussian fitting algorithm. The global search for each time point yielded a unique best-fit with χ^2 close to unity. An example 2D χ^2 contour plot for the S_{M3c}, n_F parameter pair at PF is shown in Fig. S3. The SDs for n_F, S_{M3m}, *m1* and *m2* at PF quoted in the main text were obtained by ten runs of a Monte Carlo simulation of the fitting algorithm in which the mean of each experimental parameter was replaced by a value chosen at random from a normal distribution with the experimental SD for that parameter.

The fitting procedure described in the previous paragraph failed at very early and late time points at which the value of n_A was too small to give a reliable estimate of n_F. However the results for all other time points were consistent with about 30% of the motors in the ordered layers being in the *I* conformation (calculated by subtracting the A, P and F motors from the total) within this time range. Since it is very unlikely that there is a sudden jump in n_I early after stimulation that is reversed in late relaxation, we extrapolated this conclusion to diastole, i.e. we also assumed that 30% of the motors in the ordered layers (making up almost all the layers of motors in diastole) are disordered in diastole. This assumption is supported by the approximate fit between the observed and calculated changes in the total intensity of the M3 reflection (I_{M3}, Fig. 6*B*). However the dependence of I_{M3} on other structural parameters, including the systematic displacements of the three motor layers in each ca 44-nm helical repeat from the ideal 14.5-nm spacing in the resting or diastolic states that is linked to the presence of the so-called forbidden reflections M1, M2, M4 etc and is reduced on activation (15), means that a more extensive analysis and a more

complicated model would be required for a definitive interpretation of I_{M3}. For that reason I_{M3} was excluded from the fitting procedure described above.

To define S_{M3m} in the above calculations, the axial periodicity of the myosin motors in the C-zone (S_{M3c}, Fig. 6*F*) was constrained to be 2% shorter than that in the P- and D-zones (S_{M3pd}), as explained in the main text. The 2% difference was estimated as follows. S_{M3c} at PF was obtained from the observed S_{M3}, when only the C-zone contributes to the M3 reflection. S_{M6c} at PF was calculated as $S_{M3c}/2$ under the assumption that, in a given zone, motors index on the local backbone periodicity. S_{M6pd} at PF was then estimated from the experimental value of S_{M6} assuming that layers 1 to 46 of the myosin filament contribute to the M6 reflection, i.e. the same as the layers contributing to the M3 reflection in diastole. This assumption is supported by the observation that, within the precision of the measurements, $S_{M3} = 2^* S_{M6}$ in diastole (Table S1). As expected from the presence of two distinct periodicities differing by 2%, the profile of the M3 reflection in diastole calculated from the model is more complex than that described here in terms of LA, MA and HA peaks (Fig. 5*C*); a series of additional peaks should be present on the low-angle side with intensity less than 10% of that of the main peak. Such peaks would not be experimentally detectable in the present protocol and preparation with the available signal:noise.

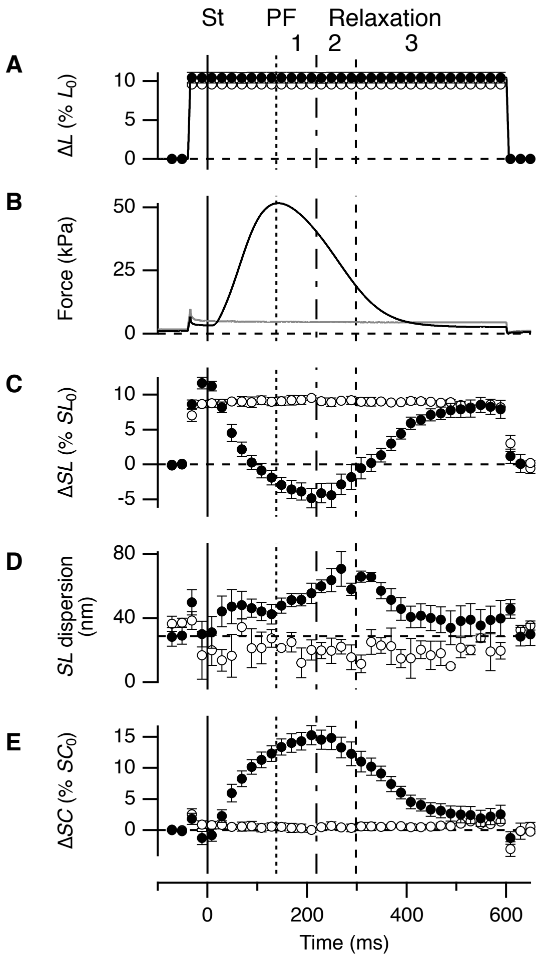

Fig. S1. Mechanical response to a stretch in electrically-paced and unstimulated trabeculae. Black symbols, electrically-paced trabeculae (mean ± SEM, *N*=6 trabeculae); open symbols, unstimulated trabeculae (*N* =2 trabeculae). Vertical continuous line, stimulus (St, t=0); vertical dotted line, peak force (PF); vertical dot-dashed line, end of phase 1 of relaxation; vertical dashed line, end of phase 2 of relaxation; horizontal dashed line, zero. (*A*) Trabecular length change (Δ*L*), relative to the initial length of the trabecula (L_0). A 10%-ramp stretch complete in 5 ms was applied 40 ms before the stimulus. (*B*) Force response in electrically-paced (black) and unstimulated trabeculae (grey). (*C*) Sarcomere length change after the stretch (Δ*SL*) relative to the initial value (SL_0) in electrically-paced (filled circles) and unstimulated trabeculae (open circles). Note that Δ*SL* in unstimulated trabeculae reaches a maximum 40 ms after the stretch and then remains constant, suggesting that the viscoelastic behaviour is a property of the sarcomere rather than the series compliance. (*D*) Sarcomere length dispersion estimated from the width of a Gaussian fit to the axial intensity distributions of the 2nd order sarcomere reflections. (*E*) Changes in length of the series compliance (Δ*SC*), relative to the value before the stretch (SC_0), calculated as ($\Delta L/L_0$)-($\Delta SL/SL_0$). Note that the transient increase in Δ*SC* after the stretch is consistent with the presence of a viscoelastic element in the sarcomere.

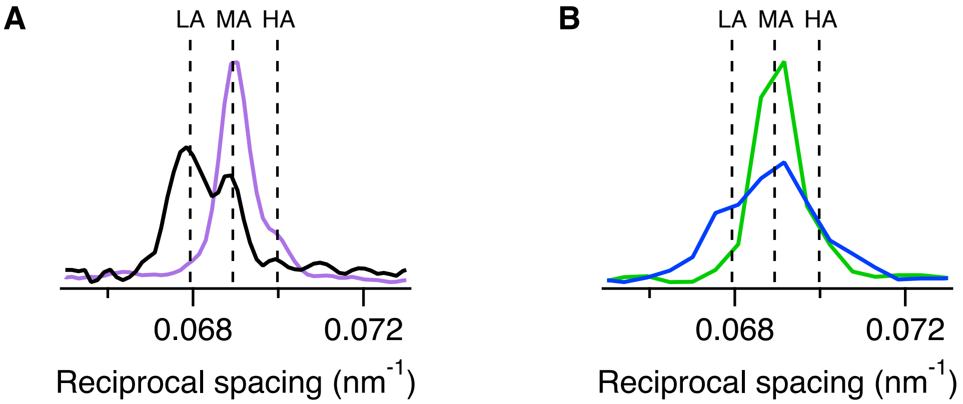

Fig. S2. Axial profiles of the M3 reflection in demembranated and intact trabeculae. (*A*) M3 profiles recorded with the FReLoN detector (sample-to-detector distance, 1.6 m) from relaxed ([Ca^{2+}]=1 nM, purple) and maximally activated ([Ca^{2+}]=20 μM, black, active force 95 kPa, as in Fig. 5*I*) demembranated trabeculae. Data added from two trabeculae. Total exposure time, 40 ms. (*B*) M3 profiles recorded with the Pilatus detector (sample-to-detector distance, 3.2 m) from electrically-paced intact trabeculae in diastole (green) and at peak force (blue), as in Fig. 5*C*, *F*. Data added from 6 trabeculae. Total exposure time, 57.63 ms. Vertical dashed lines mark the position of low- (LA), middle- (MA) and high-angle (HA) peaks in relaxed trabeculae (A) and in diastole (B).

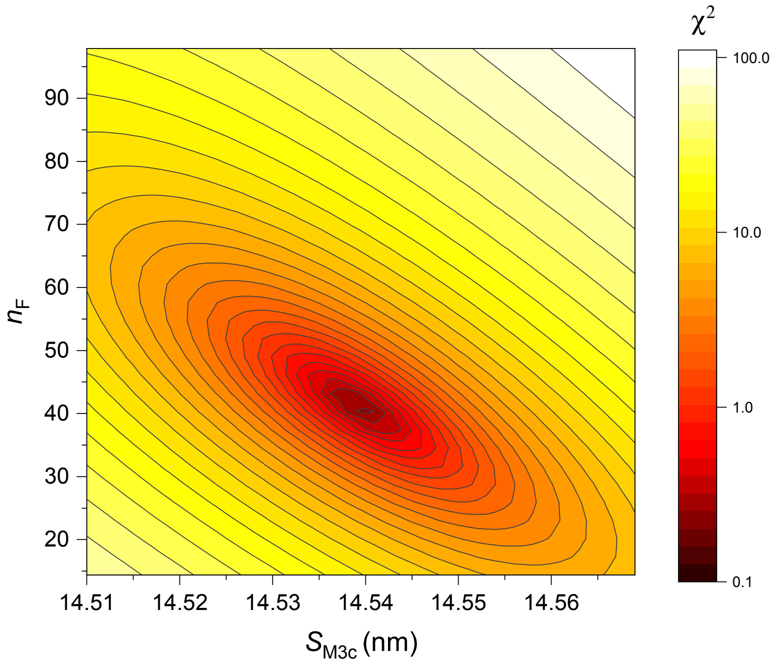

Fig. S3. Example χ^2 surface for two model parameters around the global minimum at peak force. The 4D-distribution of χ^2 was calculated as a function of the four model parameters (S_{M3c}, n_F, and the first and last contiguous layer of ordered motors). The Fig. shows the χ^2 at PF as a function of n_F and S_{M3c} (global minimum χ^2 = 0.23; S_{M3c} = 14.54 nm; n_F = 40) for contiguous layers 6 to 29.

Table S1. Mechanical and structural parameters in intact and demembranated trabeculae. Force, sarcomere length (*SL*), intensity ratio of the equatorial reflections (I_{11}/I_{10}), axial spacings of M6 (S_{M6}) and M3 (S_{M3}) reflections, and intensity of ML1 (I_{ML1}) in intact trabeculae in diastole and at peak force and in demembranated trabeculae in relaxation ([Ca^{2+}]=1 nM, pCa 9.0), active isometric contraction ([Ca^{2+}]=20 µM, pCa 4.7) and rigor (pCa 9.0, no ATP), 27°C. Force, SL and I_{11}/I_{10} are mean ± SEM. *n*, number of trabeculae. S_{M6}, S_{M3} and I_{ML1} added from *n* trabeculae.

[a]SL in rigor measured in one trabecula.

[b]I_{ML1} not measurable at pCa 4.7.

	n	Force (kPa)	*SL* (µm)	I_{11}/I_{10}	S_{M6} (nm)	S_{M3} (nm)	I_{ML1}
Diastole	6	2.2 ± 0.2	2.17 ± 0.01	0.25 ± 0.01	7.245	14.488	1
Peak Force	6	50.4 ± 4.1	1.89 ± 0.02	0.92 ± 0.04	7.320	14.524	0.23
pCa 9	4	0	2.13 ± 0.02	0.23 ± 0.03	7.233	14.479	1
pCa 4.7	2	94.6 ± 1.0	1.85 ± 0.18	1.47 ± 0.01	7.374	14.658	[b]
Rigor pCa 9	4	4.5 ± 0.1	2.05[a]	2.39 ± 0.38	7.345	14.651	0.33

Table S2. Half-times of mechanical, structural and model parameters during force development Half times ($t_{1/2}$, mean ± SD, with respect to the stimulus) to PF calculated by fitting a straight line to the points around the half-change of experimental data in Fig. 2 and model parameters in Fig. 6. A_{ML1} is calculated as $(I_{ML1})^{1/2}$ and is proportional to the number of helical motors.

	$t_{1/2}$ (ms)
Force	65.4 ± 7.0
SL	51.1 ± 5.4
I_{11}/I_{10}	65.8 ± 1.6
S_{M6}	59.1 ± 1.9
I_{ML1}	63.9 ± 8.7
A_{ML1}	65.4 ± 13.1
S_{M3c}	92.8 ± 3.5
n_F	73.4 ± 4.0
n_A	62.5 ± 6.6
n_I	77.8 ± 9.0

Movie S1 (separate file). X-ray diffraction movie of the sarcomere reflections during the heartbeat. Ultra-small angle X-ray patterns in the spacing range ~1 to 0.25 μm (2nd to 8th order of the sarcomeric repeat), collected from one electrically-paced trabecula at 20 ms intervals with the Pilatus detector at 31m. Equivalent full beam exposure time per frame, 1.5 ms.

Movie S2 (separate file). X-ray diffraction movie of the myosin-based reflections during the heartbeat. Small angle X-ray patterns with meridional periodicities in the range 70-6 nm collected at 20 ms intervals with the Pilatus detector at 3.2 m. The black horizontal lines are gaps between detector modules, with the equatorial and the myosin-based ML1/M1 and M2 reflections in the middle module. The M3 and M6 reflections are near the edges of the outer modules. Data added from three electrically-paced trabeculae. Equivalent full beam exposure time per frame, 30 ms.

SI References

1. Luther PK*, et al.* (2008) Understanding the organisation and role of myosin binding protein C in normal striated muscle by comparison with MyBP-C knockout cardiac muscle. *J. Mol. Biol.* 384(1):60-72.
2. Reconditi M*, et al.* (2017) Myosin filament activation in the heart is tuned to the mechanical task. *Proc. Natl. Acad. Sci. U.S.A.* 114(12):3240-3245.
3. Reconditi M*, et al.* (2011) Motion of myosin head domains during activation and force development in skeletal muscle. *Proc. Natl. Acad. Sci. U.S.A.* 108(17):7236-7240.
4. Piazzesi G*, et al.* (2007) Skeletal muscle performance determined by modulation of number of myosin motors rather than motor force or stroke size. *Cell* 131(4):784-795.
5. Piazzesi G*, et al.* (2002) Mechanism of force generation by myosin heads in skeletal muscle. *Nature* 415(6872):659-662.
6. Brunello E*, et al.* (2007) Skeletal muscle resists stretch by rapid binding of the second motor domain of myosin to actin. *Proc. Natl. Acad. Sci. U.S.A.* 104(50):20114-20119.
7. Reconditi M (2006) Recent improvements in small angle X-ray diffraction for the study of muscle physiology. *Rep. Prog. Phys.* 69(10):2709-2759.
8. Woodhead JL*, et al.* (2005) Atomic model of a myosin filament in the relaxed state. *Nature* 436(7054):1195-1199.
9. Zoghbi ME, Woodhead JL, Moss RL, & Craig R (2008) Three-dimensional structure of vertebrate cardiac muscle myosin filaments. *Proc. Natl. Acad. Sci. U.S.A.* 105(7):2386-2390.
10. Irving M (2017) Regulation of contraction by the thick filaments in skeletal muscle. *Biophys. J.* 113(12):2579-2594.
11. Linari M*, et al.* (2015) Force generation by skeletal muscle is controlled by mechanosensing in myosin filaments. *Nature* 528(7581):276-279.
12. Huxley HE, Reconditi M, Stewart A, & Irving T (2006) X-ray interference studies of crossbridge action in muscle contraction: evidence from muscles during steady shortening. *J. Mol. Biol.* 363(4):762-772.
13. Reconditi M*, et al.* (2004) The myosin motor in muscle generates a smaller and slower working stroke at higher load. *Nature* 428(6982):578-581.
14. Pinzauti F*, et al.* (2018) The force and stiffness of myosin motors in the isometric twitch of a cardiac trabecula and the effect of the extracellular calcium concentration. *J. Physiol.* 596(13):2581-2596.
15. Huxley HE & Brown W (1967) The low-angle x-ray diagram of vertebrate striated muscle and its behaviour during contraction and rigor. *J. Mol. Biol.* 30(2):383-434.
